# Progression of naive intraepithelial neoplasia genome to aggressive squamous cell carcinoma genome of uterine cervix

**DOI:** 10.18632/oncotarget.2981

**Published:** 2015-01-23

**Authors:** Seung-Hyun Jung, Youn Jin Choi, Min Sung Kim, In-Pyo Baek, Sung Hak Lee, Ah Won Lee, Soo Young Hur, Tae-Min Kim, Sug Hyung Lee, Yeun-Jun Chung

**Affiliations:** ^1^ Department of Microbiology, College of Medicine, The Catholic University of Korea, Seoul 137-701, South Korea; ^2^ Department of Pathology, College of Medicine, The Catholic University of Korea, Seoul 137-701, South Korea; ^3^ Department of Integrated Research Center for Genome Polymorphism, College of Medicine, The Catholic University of Korea, Seoul 137-701, South Korea; ^4^ Department of Hospital Pathology, College of Medicine, The Catholic University of Korea, Seoul 137-701, South Korea; ^5^ Department of Obstetrics/Gynecology, College of Medicine, The Catholic University of Korea, Seoul 137-701, South Korea; ^6^ Department of Medical Informatics, College of Medicine, The Catholic University of Korea, Seoul 137-701, South Korea

**Keywords:** Uterine cervix cancer, Cervical intraepithelial neoplasia, Cervical squamous cell carcinoma, Mutation, Copy number alteration

## Abstract

Although cervical intraepithelial neoplasia (CIN) is considered a neoplasia, its genomic alterations remain unknown. For this, we performed whole-exome sequencing and copy number profiling of three CINs, a microinvasive carcinoma (MIC) and four cervical squamous cell carcinomas (CSCC). Both total mutation and driver mutation numbers of the CINs were significantly fewer than those of the MIC/CSCCs (*P* = 0.036 and *P* = 0.018, respectively). Importantly, *PIK3CA* was altered in all MIC/CSCCs by either mutation or amplification, but not in CINs. The CINs harbored significantly lower numbers of copy number alterations (CNAs) than the MIC/CSCCs as well (*P* = 0.036). Pathway analysis predicted that the MIC/CSCCs were enriched with cancer-related signalings such as cell adhesion, mTOR signaling pathway and cell migration that were depleted in the CINs. The mutation-based estimation of evolutionary ages identified that CIN genomes were younger than MIC/CSCC genomes. The data indicate that CIN genomes harbor unfixed mutations in addition to human papilloma virus infection but require additional driver hits such as *PIK3CA, TP53, STK11* and *MAPK1* mutations for CSCC progression. Taken together, our data may explain the long latency from CIN to CSCC progression and provide useful information for molecular diagnosis of CIN and CSCC.

## INTRODUCTION

Uterine cervical cancer is the third most common malignancy in women worldwide, and is responsible for 10–15% of cancer-related deaths in women [1]. Human papilloma virus (HPV) is the single most crucial causative agent in the development of cervical cancer [2]. Cervical intraepithelial neoplasia (CIN), also known as cervical dysplasia, is premalignant lesions of the cervical squamous cell carcinoma (CSCC) that shows abnormal growth of squamous cells in the cervix epithelium. The CIN consists of CIN1 (mild dysplasia confined to the basal 1/3 of the epithelium), CIN2 (moderate dysplasia confined to the basal 2/3 of the epithelium) and CIN3 (severe dysplasia that spans more than 2/3 of the epithelium or carcinoma *in situ* (CIS)) [3]. Although CIN phase is generally stable, it can progress to CSCC after a long latency (10~20 years) [2]. The serial but latent progression of CIN to CSCC indicates that other events besides HPV infection may occur during the progression.

Cervical CIN and CSCC are known to share chromosomal abnormalities in common, although the prevalence in CSCC is higher [4, 5]. As for tumor cell clonality, most of the CIN1 and CIN2, and all of the CIN3 display monoclonality [6]. Also, miRNA expression pattern displays a distinct separation between CIN and normal cervical epithelium [7]. These data indicate that CIN lesions, especially CIN 2 and 3 are true neoplasia, not simple tissue reactions to HPV infection and that CIN harbors genetic and epigenetic aberrations as well as HPV infection.

For a comprehensive elucidation of genetic alterations in cervical cancers, genomes of CSCC were studied using whole-exome sequencing analysis [8]. They identified recurrent somatic mutations of *PIK3CA, PTEN, STK11, EP300, FBXW7, HLA-B, MAPK1* and *NFE2L2* in CSCC [8]. However, this study did not include CIN tissues and we still do not know the mutational landscape of CIN genomes. Given the evidence suggesting that differences may exist between CIN and CSCC, we hypothesize that progression may be mediated by subpopulation selection or by acquisition of additional alterations, including gene mutations or chromosomal alterations. In this study, we analyzed cervical CIN, microinvasive carcinoma (MIC) and CSCC by whole-exome sequencing and array-comparative genomic hybridization (array-CGH) and found that CIN genomes harbored fewer mutations (especially fewer driver mutations) and copy number alterations (CNAs), suggesting that additional genomic alterations might burst onto the CIN genome at the final stage of CIN progression to CSCC or an early stage of CSCC.

## RESULTS

### Whole-exome sequencing of CIN and CSCC genomes

**Figure 1:**
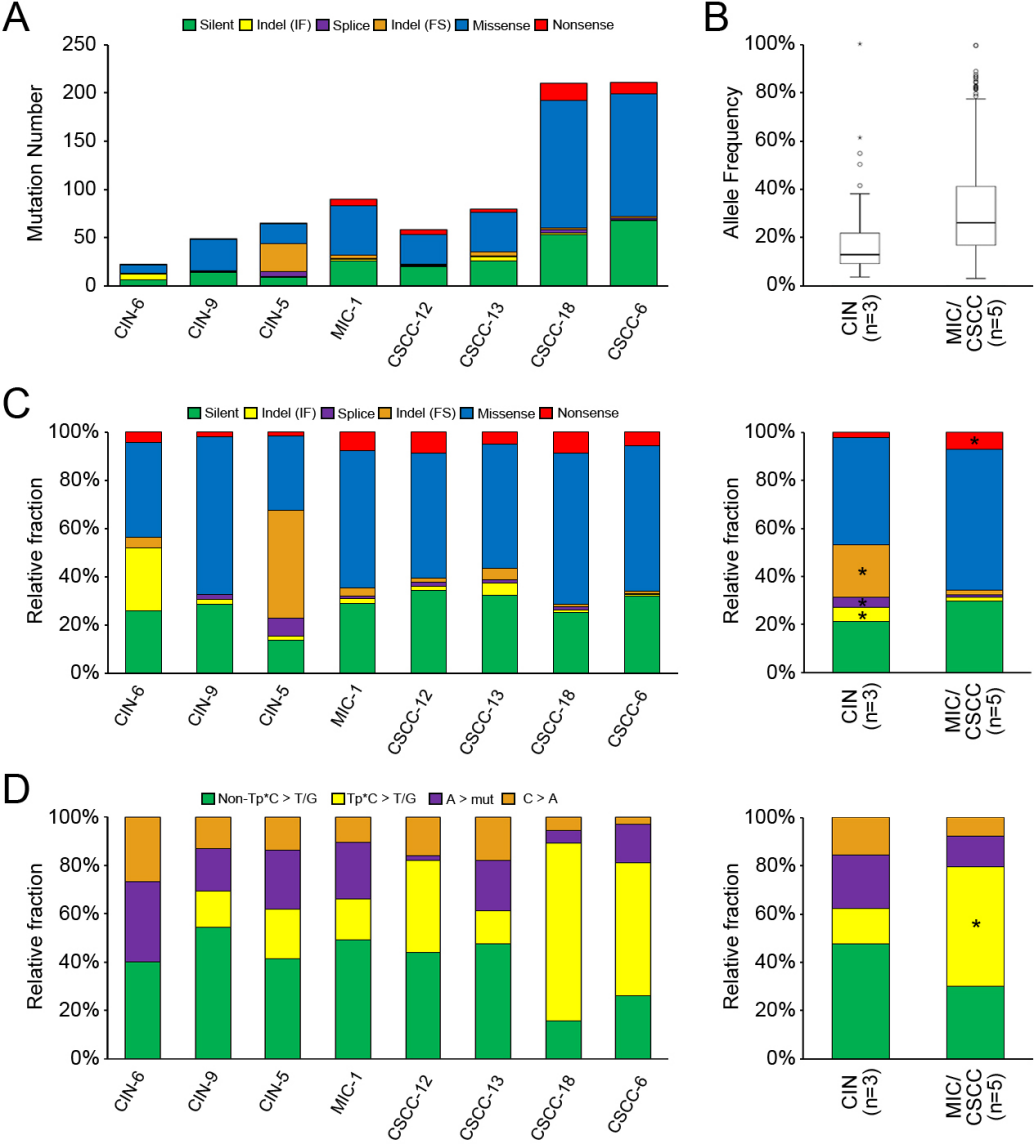
The mutational features of eight cervical neoplasia genomes **(A)** The numbers of somatic mutations are shown with the six functional categories indicated in the inset. **(B)** Mutant allele frequencies (y-axis) are shown for the total number of mutations observed in the CIN and MIC/CSCC genomes with significant difference between them (*P* = 8.5 × 10^−14^). **(C)** Relative fraction of six functional categories. Relative fraction (y-axis) for each case is shown in left panel. Relative fraction for CIN and MIC/CSCC genomes is shown in the right panel. Asterisk shows the relative enrichment (> 3 fold changes) of the corresponding mutation categories. **(D)** The point mutations are classified according to both base context and sequence changes. Relative fraction of sequence-based mutation categories (y-axis) for each case is shown in the left panel. Relative fraction for CIN and MIC/CSCC genomes is shown in the right panel. Asterisk shows the relative enrichment (> 3 fold changes) of the corresponding mutation categories.

To find genomic differences between CIN and CSCC, a total of eight cervical neoplasia genomes (three CINs, one MIC, and four CSCC genomes) were analyzed in this study (Table 1). For the comparison with CIN, both CSCC and MIC were regarded as one group in all of the analyses. First, we performed whole-exome sequencing of the eight cervix neoplasia and their matched normal genomes. Coverage of depth was median of 70X (61–80X) for tumor samples and 68X (60–85X) for normal samples (Supporting Information Table S1). Using the MuTect algorithm [9] and the SomaticIndelDetector [10], we identified 23–211 point mutations and indels per sample (median of 73 somatic variants) (Figure 1A, Supporting Information Table S2). Mutation numbers of the MIC/CSCC genomes (58–211; median of 90 mutations) were significantly higher than those of the CIN genomes (23–65; median of 49 mutations) (*P* = 0.036, Table 2).

**Table 1:** Clinocopathologic features of the patients

Case	Age	Diagnosis	TNM stage	HPV type	Treatment
CIN-5	35	CIS	TisN0M0	16, 52	LEEP
CIN-6	46	CINIII	TisN0M0	31, 61	LEEP
CIN-9	38	CIS	TisN0M0	33	LEEP
MIC-1	38	MIC	T1aN0M0	16	LEEP
CSCC-6	43	CSCC	T1b1N0M0	16, 52	Radical hysterectomy
CSCC-12	43	CSCC	T2bN1M0	16	Radical hysterectomy
CSCC-13	48	CSCC	T2a2N0M0	16, 70	Radical hysterectomy
CSCC-18	49	CSCC	T2bN0M0	16	Radical hysterectomy

CIN: cervical intraepithelial neoplasia, CIS: carcinoma *in situ*, MIC: microinvasive carcinoma, CSCC: cervical squamous cell carcinoma, LEEP: loop electrical excision

**Table 2:** Summary of comparison data between CIN and MIC/CSCC genomes

	CIN vs. MIC/CSCC
Somatic mutation number	CIN < MIC/CSCC (*P* = 0.036)
Mutation allele frequency	CIN < MIC/CSCC (*P* = 8.5 × 10^−14^)
Inferred evolutionary age	CIN < MIC/CSCC (*P* = 0.018)
Driver mutation number	CIN < MIC/CSCC (*P* = 0.018)
Number of CNA	CIN < MIC/CSCC (*P* = 0.036)
Length of CNA	CIN < MIC/CSCC (*P* = 0.01)

As for mutant allele frequencies (MAFs) of the point mutations, mutations of the MIC/CSCCs had significantly higher allele frequencies than those of the CINs (mean MAF 0.30 in MIC/CSCCs and mean MAF 0.17 in CINs, *P* = 8.5 × 10^−14^) (Figure 1B, Table 2). Based on these MAFs, we inferred evolutionary ages of the eight cervical neoplasia genomes. We adopted an evolutionary model that used somatic mutations as molecular clocks to estimate the evolutionary ages of cancer genomes. We inferred the relative timing between the birth of a founder cell (i.e., the cell of origin for tumor initiation) and the emergence of the last common ancestor before the last cycle of clonal amplification for the eight cervical genomes. The numbers of clonal mutations in MIC/CSCC genomes were 18 to 190 that gave conservative estimates of evolutionary ages of 720 to 7600 cell cycles (i.e., the cell cycles required between the emergence of the founder cell and the last common ancestor). For CIN, the genomes showed 5 to 7 clonal mutations corresponding to 200 to 280 cell cycles of evolutionary ages. The evolutionary ages estimated from the MIC/CSCC genomes were significantly longer than those estimated from the CIN genomes (*P* = 0.018, Table 2). Details of the estimated evolutionary ages are available in Supporting Information Figure S1. With respect to the mutation spectra, frameshift indels, inframe indels, and splicing variants were relatively enriched (> three fold changes) in the CIN genomes, whereas nonsense mutations were relatively enriched in the MIC/CSCC genomes (Figure 1C). In terms of sequence composition of the point mutations, Tp*C to T/G mutation was relatively enriched in the MIC/CSCC genomes (Figure 1D). Of note, the relative frequencies of Tp*C to T/G mutation were positively correlated with the number of mutations (R^2^ = 0.79).

### Copy number alterations and their distribution in CIN and CSCC genomes

**Figure 2:**
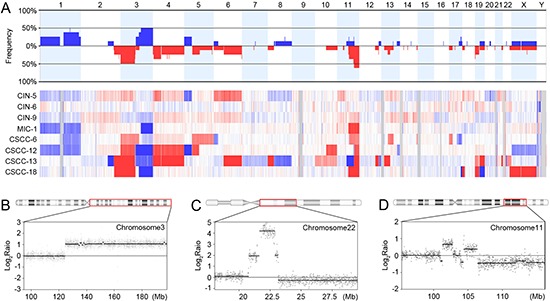
Copy number profiles, amplification and chromothripsis **(A)** Frequencies (y-axis) of copy number gains and losses across the whole genomes of the eight cervical neoplasia genomes (upper panel) and their heatmap for probe-level signal intensities (lower panel). Blue denotes the copy number gains and the red denotes the copy number losses. **(B)** Amplification on chromosome 3q21.2-q29 in MIC-1, where the *PIK3CA* and *SOX2* oncogenes are located. **(C)** Amplification on chromosome 22q11.21-q11.22 in CSCC-13, where the *MAPK1* and *BCR* oncogenes are located. **(D)** The complex recombination event (chromothripsis) on chromosome 11q22.1-q25 in CSCC-12. X-axis represents the genomic location and y-axis represents signal intensities on the log_2_ scale.

We next performed array-CGH for the eight cervical neoplasia genomes with their matched normal genomes as references. A total of 92 CNAs were identified in the eight samples (Figure 2A, Supporting Information Table S3). The MIC/CSCC genomes harbored significantly higher numbers of CNAs than the CIN genomes (median of 18 vs. 4 CNAs, respectively, *P* = 0.036). The MIC/CSCC genomes harbored significantly longer CNAs (655 Mb-sized regions per MIC/CSCC genome, ranged 368–962 Mb) than the CIN genomes (93 Mb, ranged 1.7–275 Mb) (*P* = 0.01, Table 2).

An MIC (MIC-1) harbored amplification on 3q21.2-q29 that contained both *PIK3CA* and *SOX2* oncogenes (Figure 2B). Also, a CSCC (CSCC-13) harbored amplification on 22q11.21-q11.22 that contained both *MAPK1* and *BCR* oncogenes (Figure 2C). In a CSCC (CSCC-12), a potential chromothripsis showing complex recombination events was observed on chromosome 11q22.1-q25, where *BIRC3, YAP1, MMP* and *Caspase* genes reside (Figure 2D).

Of the 92 CNAs, 10 were identified recurrently (> 3 cases) (Supporting Information Table S4). Several cancer-related genes *PIK3CA, EVI1, SOX2, ETV5, ARNT, SETDB1, APH1A, CHD1L, PRKCI* and *ELK4* were located within the recurrently gained regions, while the *VHL, XPC, MLH1, CTNNB1, FOXP1, STK11* and *PTPN13* genes were located within the recurrently deleted regions. Recurrent copy number losses on 2q37.1, 4p, 6q27 and 19p13.3 were detected in both CINs and MIC/CSCCs, while recurrent copy number gains on 1q and 3q as well as losses on 3p, 4q21.3, 4q22.3-q23 and 11q were found in the MIC/CSCCs, but not in the CINs (Supporting Information Table S4).

### Driver mutations and pathways of cervical neoplasia genomes

**Figure 3:**
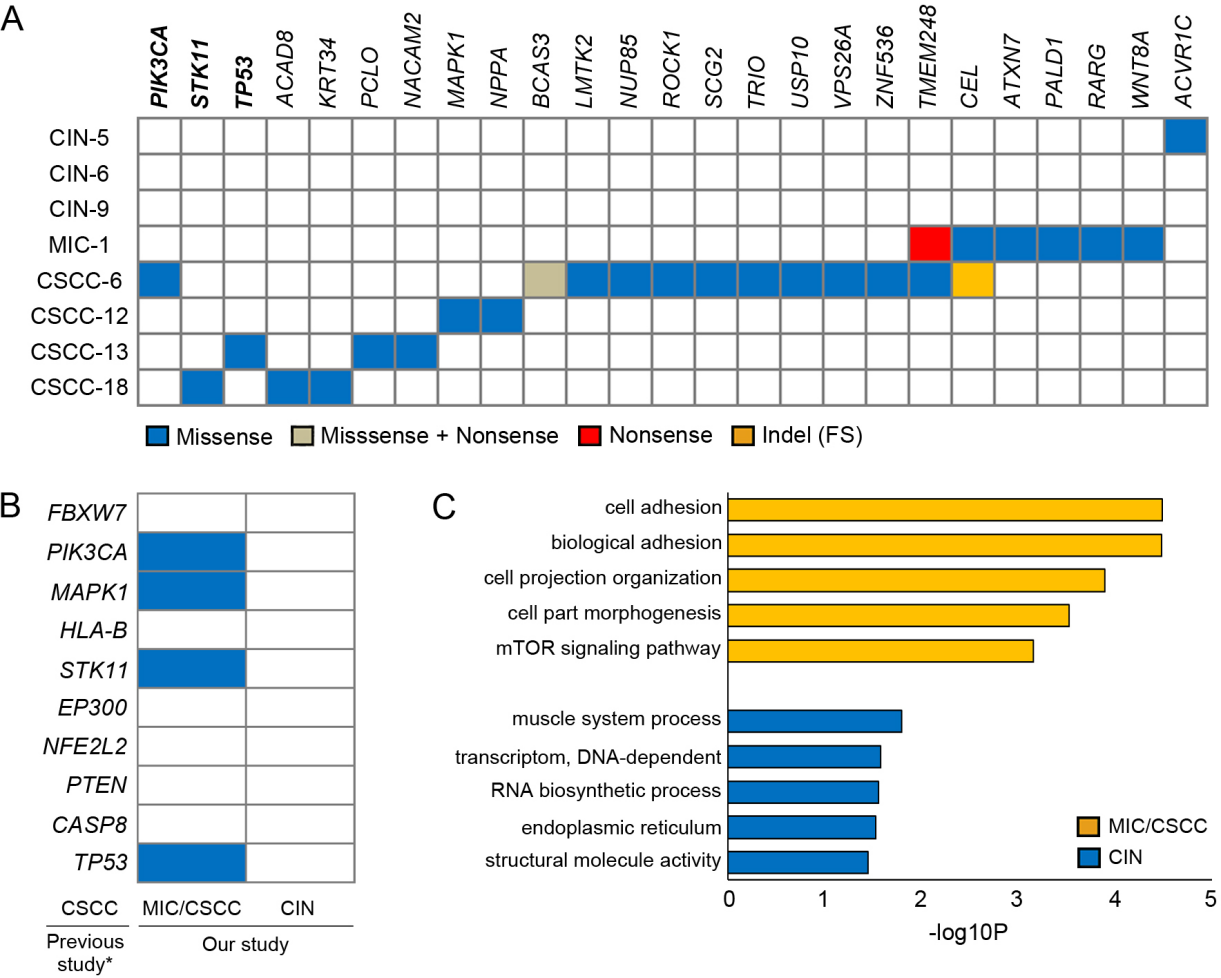
Driver mutations and pathway analyses **(A)** The 25 candidate driver mutations identified by the CHASM analysis with the four functional categories indicated in the inset. *PIK3CA, STK11* and *TP53* genes (bold) were overlapped with both cancer Gene Census and the cervix top 20 genes in the COSMIC database. **(B)** Comparison of our candidate drivers with the top ten significant mutations in previous study [8]. Asterisk indicates the Ojesina et al's report [8]. Blue boxes represent the mutations detected in this study. **(C)** The results of the DAVID pathway analyses. Top five functional clusters are shown for the MIC/CSCC and CIN genomes. X-axis represents the log-transformed *P*-values.

In order to identify the putative driver mutations that may be causally implicated in uterine cervical neoplasia development, we performed the CHASM analysis [11], which predicted driver mutations (Figure 3A). All (n = 25) of the predicted driver mutations except one were detected in the MIC/CSCC genomes. All of the MIC and CSCC harbored more than two drivers (2 to 12 drivers) per genome. To the contrary, two CINs (CIN-6 and CIN-9) did not harbor any of the drivers and a CIN (CIN-5) harbored one driver mutation. There was a significant difference in the driver mutation numbers between the CINs and MIC/CSCCs (*P* = 0.018, Table 2). Of the 25 driver genes, both *TMEM248* and *CEL* mutations were detected recurrently in two cases, while the other 23 gene mutations were singletons. Three of them (*PIK3CA, STK11* and *TP53*) were overlapped with both cancer Gene Census [12] and cervix top 20 genes in the COSMIC database. When we compared our candidate drivers with the top ten significant mutations reported in the recent study by Ojesina et al [8], four of them (*PIK3CA, MAPK1, STK11* and *TP53*) were overlapped (Figure 3B). As for the CNAs, seven out of the ten recurrent CNA regions identified in our study were identified in the previous report [8].

Of note, all of the MIC/CSCCs showed genetic alterations in *PIK3CA* gene either by CNAs or somatic mutations (Supporting Information Figure S2). A CSCC (CSCC-18) harbored both missense mutation and single copy deletion in *STK11* gene (Supporting Information Figure S3A–G), suggesting biallelic inactivation of this gene. In addition, Rho-related genes *ROCK1* and *TRIO* that play important roles in cell adhesion, motility and metastasis [13] were mutated in the MIC/CSCCs.

Next, to investigate pathway-level relationship of the individual mutations, we performed the DAVID analysis (http://david.abcc.ncifcrf.gov) and found that mutated genes in the MIC/CSCCs were significantly associated with many tumorigenesis-related gene functions, including ‘cell adhesion’ (*P* = 3.3 × 10^−5^), ‘mTOR signaling pathway’ (*P* = 7.0 × 10^−4^), and ‘cell migration’ (*P* = 0.01) (Figure 3C). According to the DAVID analysis, there was no significantly enriched cancer-related functional gene sets in the CIN genomes. Further details of the DAVID analysis are available in Supporting Information Table S5.

### Mutations co-occurred with CNAs

We detected 114 mutations that co-occurred with CNAs in the same patients (Supporting Information Table S6). There was a significant difference in the number of mutations that co-occurred with CNAs between the CINs (0–7; median of 1 mutations) and the MIC/CSCCs (13–31; median of 16 mutations) (*P* = 0.018). Among them, nine genes (*STK11, MAML2, ABL2, PRDM16, PRKAR1A, ACAD8, PIK3R1, NPPA* and *NUP85*) were overlapped with the cancer Gene Census [12] or with the putative driver genes estimated by the CHASM analysis as mentioned above. Five mutations (*ABL2, PRDM16, PRKAR1A, NPPA* and *NUP85*) co-occurred with copy number gains, while four gene mutations (*STK11, MAML2, ACAD8* and *PIK3R1*) co-occurred with copy number losses (Supporting Information Figure S3A–G). All of the nine gene mutations were found in the MIC/CSCCs, but not in the CINs.

## DISCUSSION

Although CIN is known to be a true neoplasm and frequently progresses to CSCC (2, 6), genetic alterations of CIN are largely unknown. Such lack of genetic data led us to analyze CIN and CSCC genomes in this study. The aim of this study was twofold. First, we attempted to disclose somatic mutations in entire coding genes and genome-wide CNAs of both CIN and CSCC. Second, we attempted to disclose genomic differences between CIN and CSCC that may drive CIN to CSCC. We found that CIN harbored fewer somatic mutations and CNAs than CSCC. More importantly, CIN harbored much fewer driver mutations than CSCC. Our data indicate that CINs have quantitatively and qualitatively naive genomes, which may need further genetic hits to develop CSCC genomes. We also defined potential driver mutations for cervical cancer development and found that mutated genes in the MIC/CSCCs were significantly associated with essential tumorigenesis-related functions such as cell adhesion and migration.

Using the CHASM analysis, we identified 25 candidate driver gene mutations, all except one of which were detected exclusively in the MIC/CSCCs, but not in the CINs. The only one driver mutation identified in a CIN was a missense mutation (p.T481A) in *ACVR1C* that encodes a type I receptor for the TGF-β signaling. *ACVR1C* mutations have been detected in CSCC by neither our study nor the previous study [8]. The *ACVR1C* p.T481A mutation has not been reported in any other tumors, either. These data indicate that *ACVR1C* mutation identified in the CIN may not play a driving role for tumorigenesis in that case, suggesting there is virtually no driver gene mutation in the CIN data set. Moreover, the CSCCs had significantly higher MAF than the CINs (*P* = 8.5 × 10^−14^). Together, these data suggest that the somatic mutations may not only be accumulated, but also be clonally fixed at the final stage of CIN progression to CSCC or an early stage of CSCC.

Up to now, there has been only one whole-exome level mutation study on CSCC [8]. Mutations in four genes (*PIK3CA, TP53, STK11* and *MAPK1*) were recurrently identified as driver mutations by both our and previous studies [8]. *PIK3CA* mutations have been reported in 14 to 38% of CSCC [8, 14, 15]. *PIK3CA* is amplified in various cancers and up to 70% of the CSCCs are known to harbor *PIK3CA* amplification [16]. In the present study, we found that all of the MIC/CSCCs harbored either somatic mutation or amplification of *PIK3CA*. Our data suggest that *PIK3CA* alteration is an essential pathway for the transition from CIN to CSCC/MIC. A recent phase 1 clinical trial using PI3K/AKT/mTOR inhibitors showed that CSCC patients with *PIK3CA* mutation had a clinical response to the inhibitors [15]. Our study may provide further rationale for performing future studies that explore the use of PI3K inhibitors in PIK3CA-altered (mutation or CNAs) CSCC/MIC. The mutation in *STK11* (p.S216F) identified in a CSCC was the same mutation identified in the previous study [8]. Also, the p.S216F mutation was found in lung cancers [17], suggesting that the recurrent mutation may be a driver in cancer pathogenesis, including transition from CIN to CSCC.

Currently, it considers MIC as carcinoma that can only be identified microscopically with a maximum depth of 5.0 mm and maximum width of 7.0 mm [18]. Patients with MICs have relatively lower risk for metastasis and recurrent cancers than those with overt CSCC [19]. In this study, we observed that the MIC showed an intermediate level of mutation numbers in between CIN and CSCC (median of 90, 49 and 145 mutations, respectively) and had a significantly lower MAF than CSCC (*P* = 1.3 × 10^−13^). Although we analyzed only one MIC, our data suggest a possibility that mutational burdens of cervical neoplasia (CIN < MIC < CSCC) might be comparable to their clinical aggressiveness.

The mutation-based estimation of evolutionary ages identified that the time interval from the initiation of CSCC/MIC to the emergence of the last common ancestor was significantly longer than that of CIN. These data indicate that, in evolutionary aspect, CIN is younger than MIC/CSCC, thus suggesting that cervical neoplasia genomes during the transition from CIN to MIC/CSCC may not stand stable at genomic levels in parallel with the discernible changes at biologic and pathologic levels [2, 3].

Chromothripsis has been observed across many cancer types [20], but it has not been identified in cervical neoplasia. In our study, we found a chromothripsis pattern on 11q22.1-q25 in a CSCC, indicating that chromothripsis occurs in cervical neoplasia as well. A prevailing view has supported early occurrences of chromothripsis in cancer evolution [20], but the issue of ‘how early’ remains unknown. Although we found chromothripsis in CSCC, not in CIN, further evaluation is required to verify whether CIN genome is genetically too young to harbor chromothripsis.

We observed a preponderance of frameshift and inframe indels in the CIN genomes and nonsense point mutations in the MIC/CSCC genomes, suggesting that cervical neoplasia genomes appear more vulnerable to different types of somatic variants during the progression. It is also possible that these genomic footprints might reflect different fixation rates of the mutation types. For example, highly deleterious events such as the frameshift mutations might be negatively selected in the early progression phase. The enrichment of Tp*C to T/G mutations in the CSCC genomes compared to the CIN genomes also indicates different mutational processes dominant in the late evolutionary phases during cervical neoplasia progression. This finding was consistent with the earlier report showing that Tp*C mutations were predominant in CSCC compared to cervical adenocarcinoma [8].

The CNA profiles in the present study were largely coherent with those in the previous studies [8, 21, 22]. Recurrent copy number losses on 4p and 6q detected in both CIN and CSCC may be involved in initiation and maintenance of cervical neoplasia, while copy number gains on 1q and 3q, and losses on 3p, 4q and 11q detected only in CSCC/MIC may be involved in the progression. As for HPV genotypes and CNAs, we could not find any association, which was not in agreement with the previous report that had shown more 3q gains in CSCC with HPV 16 than other HPV genotypes [22].

To date, little is known about the genomic landscapes of premalignant or precursor lesions of cancers such as CIN. They are so small and usually used up for histologic diagnosis and there would be little or no residual tissues for genomics researches. Moreover, even after being obtainined for research purposes, they may not be used directly and need microdissection because most premalignant lesions are small and intermixed with normal cells. For microdissection of the CIN and MIC, we used frozen sections with light hematoxylin staining, which were proven to provide enough quality and quantity of genomic DNA for both whole-exome sequencing and array-CGH (data not shown). This approach would be further useful for identifying genomic landscape of other premalignant or small lesions that are hard to analyze using conventional approaches.

In summary, our data for the first time disclosed whole-exome level mutational landscape of CIN and also found that CIN genomes harbored quantitatively and qualitatively less aggressive alterations than MIC/CSCC genomes. Also, the data suggest a systemic diagram from HPV infection to invasive cervical cancer; the first step is HPV infection and its persistency, the second step is initial mutations and CNA accumulation, and the final step is the addition of driver gene mutations. The distinguishable genomic features of CIN from CSCC provide a useful resource for understanding this latently progressing disease and identifying genomic clues for differential diagnosis and therapy options for CIN and CSCC.

## MATERIALS AND METHODS

### CIN and CSCC tissues

CIN tissues resected by the loop electrical excision procedure (LEEP) from four Korean patients were obtained from the Tissue Bank of Seoul St. Mary Hospital (Seoul, Korea). This study was approved from the institutional review board at the Catholic University of Korea, College of Medicine. Clinicopathologic features of the patients are summarized in Table 1. Following the LEEP resection, each fresh specimen was designated in a 12 o’clock fashion and cut serially. One area of the probable CIN/MIC was used for our study and resting 11 sections were sent to the diagnostic pathology unit. The selected CIN/MIC was frozen, cut and examined under microscope by a pathologist. In the diagnostic pathology unit, the same pathologist examined all of the 11 formalin-fixed, paraffin-embedded tissue sections as well as the frozen sections, and made a final diagnosis. We used the frozen tissues only when the procedure did not hamper proper diagnosis. The frozen sections were lightly stained with hematoxylin without fixation (Supporting Information Figure S4A and S4B). CIN/MIC cells and normal cells were selectively procured from hematoxylin-stained frozen sections using a 30G 1/2 hypodermic needle by microdissection as described previously [23]. Purity of the CIN/MIC cell from the microdissection was approximately 90%. To minimize DNA degradation, we finished the processes from cutting to microdissection within 30 minutes. As for CSCC, frozen tissues (approximately 0.125 cm^3^) from hysterectomy from another four patients were obtained from the Tissue Bank of Seoul St. Mary Hospital (Seoul, Korea). The CSCC specimens were evaluated for tumor purity by cutting one section for frozen section before restoring the tissue. We used the tissues only when they had > 70% tumor cell. Frozen tissues were further sliced into a fragment, which was subsequently used for genomic DNA extraction.

### Whole-exome sequencing and somatic mutations

Using genomic DNA from cervical neoplasia (CIN, MIC and CSCC) and matched normal cells, we performed exome-capture using the Agilent SureSelect Human All Exome 50Mb kit (Agilent Technologies) according to the manufacturer's instructions. DNA libraries were also constructed according to the protocol provided by the manufacturer, then whole exome-sequencing was performed using an Illumina HiSeq2000 platform to generate 101bp paired-end reads. Burrows-Wheeler aligner was used to align the sequencing reads onto the human reference genome (hg19). The aligned sequencing reads were evaluated by using Qualimap [24]. Supporting Information Table S1 shows the information of sequencing alignments (e.g., the number of reads and sequencing coverage). Processing and the management of the sequencing data were performed as described elsewhere [25, 26]. In brief, somatic genomic variants were identified by using MuTect [9] and SomaticIndelDetector [10] for point mutations and indels, respectively. ANNOVAR package was used to select somatic variants located in coding sequences and predict their functional consequences, such as silent or non-silent variants [27].

### Evolutionary models using somatic mutations

The somatic mutation-based estimation of evolutionary ages was performed as described elsewhere [26]. In brief, from the mutation rate per base pair estimated from nonsynonymous mutations in colorectal cancer genomes (5 × 10^−10^ mutations per base pair per generation) [28], we calculated the mutation rate of *r* = 50.0 × 10^6^ × 5 × 10^−10^ ≈ 0.025 per generation or cell cycle. The number of the cell divisions required to obtain *N* mutations follows a distribution with a mean of *N*/*r* [29].

### DNA copy number profiling

DNA copy number profiling was performed using Agilent Sure Print G3 Human comparative genomic hybridization (CGH) Microarray 180K. The genomic DNA from cervical neoplasia and matched normal genomes were hybridized onto the array as described elsewhere [26]. Background correction and normalization form array images were done using the Agilent Feature Extraction Software v10.7.3.1. The RankSegmentation statistical algorithm in NEXUS software v7.5 (Biodiscovery Inc.) was used to define copy number alterations of each sample. The microarray dataset has been deposited in Gene Expression Omnibus (GEO; http://www.ncbi.nlm.nih.gov/geo/) with the accession of GSE60067.

### Driver mutations and gene set analysis

In order to identify the putative driver mutations, we used the CHASM analysis program with ‘cervix’ option for cancer tissue type [11]. To investigate the gene ontology of individual mutations, we performed the DAVID analysis (http://david.abcc.ncifcrf.gov/) [30]. The ‘biological process’, ‘cellular components’, ‘molecular function’ and ‘KEGG PATHWAY’ categories were sorted by significance. The raw output for this analysis is provided in Supporting Information Table S5.

## SUPPORTING INFORMATION FIGURES AND TABLES










