# Supplementary material for: Progression of naive intraepithelial neoplasia genome to aggressive squamous cell carcinoma genome of uterine cervix
Source: Oncotarget. 2015 Jan 23;6(6):4385–93. doi: 10.18632/oncotarget.2981 (PMC4414197; doi:10.18632/oncotarget.2981)
Supplement: Supplementary file 1 [file oncotarget-06-4385-s001.pdf]

SUPPORTING INFORMATION FIGURES AND TABLES

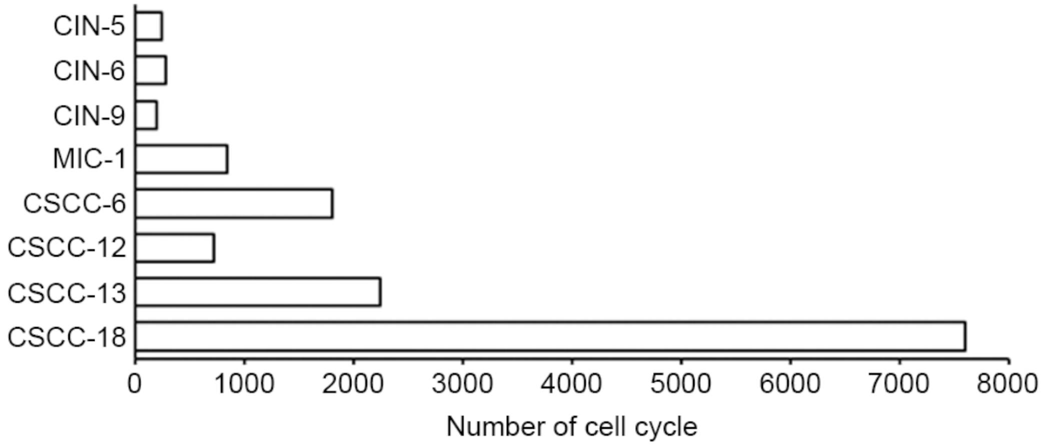

Supporting Information Figure S1: Somatic mutation-based estimation of evolutionary ages. The number of cell cycles estimated by an evolutionary model that uses somatic mutations as molecular clocks are shown for eight cervical neoplasia genomes. In this model, we used a model previously described for colorectal and pancreatic cancer genomes [16, 17]. From the mutation rate per base pair estimated from nonsynonymous mutations in colorectal cancer genomes (5×10^{-10} mutations per base pair per generation) [16], we calculated the mutation rate of $r = 50.0 \times 10^6 \times 5 \times 10^{-10} \approx 0.025$ per generation or cell cycle. The number of the cell divisions required to obtain N mutations follows a distribution with a mean of N/r [17]. Here, we were interested in the number of mutations obtained by a founder cell since its first cell division until the emergence of the last common ancestor in a single lineage. Unlike previous studies that selected clonal mutation based on the comparison between primary versus metastasis regions [16] or regional clonality in primary tumor masses [17], we selected clonally fixed mutations with manually determined mutant allele frequency cutoffs.

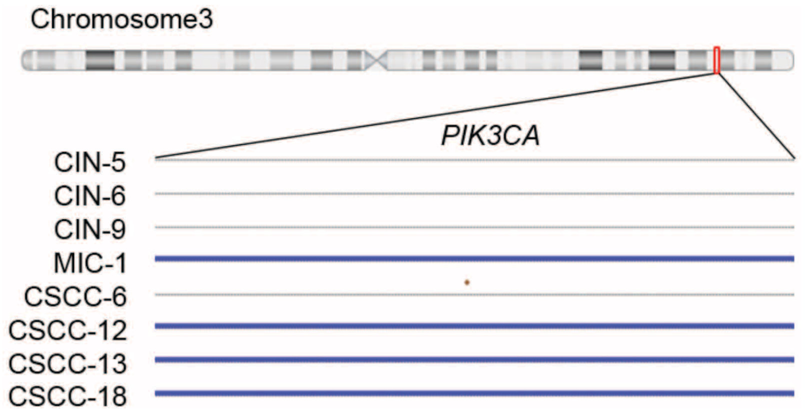

Supporting Information Figure S2: Genetic alterations in *PIK3CA* gene. All of the MIC/CSCCs showed genetic alterations in *PIK3CA* gene either by CNAs or somatic mutations. The blue denote the copy number gains and brown star represent the point mutation.

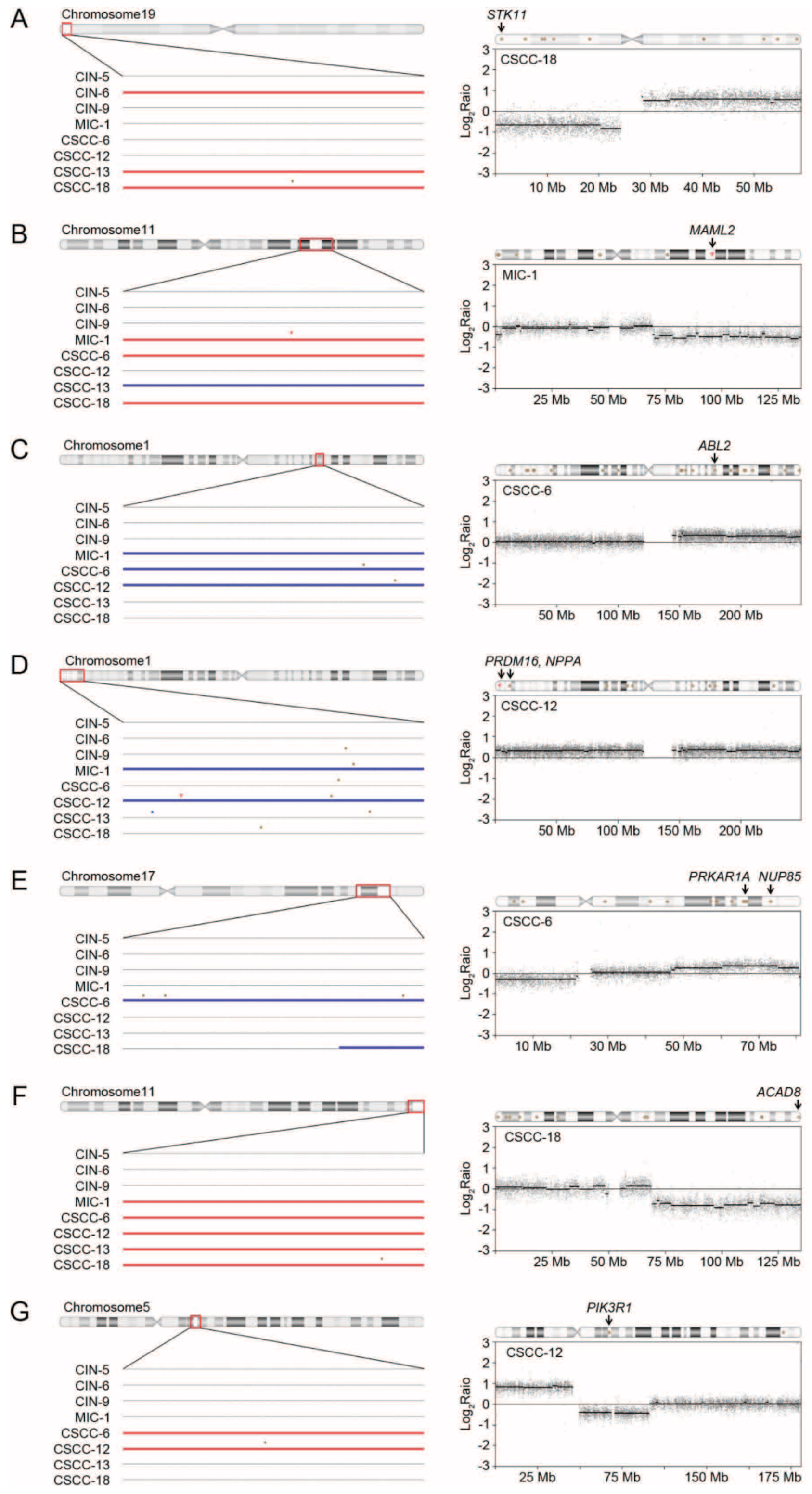

Supporting Information Figure S3: Mutations co-occurred with CNAs. Nine mutations with the cancer Gene Census or with the putative driver genes co-occurred with CNAs in the same patients. Copy number and mutation profiles in the eight cervical neoplasia genomes are shown in left panel. The blue denote the copy number gains and the red denote the copy number losses. Regarding the mutations, red triangles represent the indels and brown diamonds represent the point mutations. Examples of the co-occurrence are shown in right panel. The x-axis represents the genomic location and the y-axis represents signal intensities on the $\log_2$ scale.

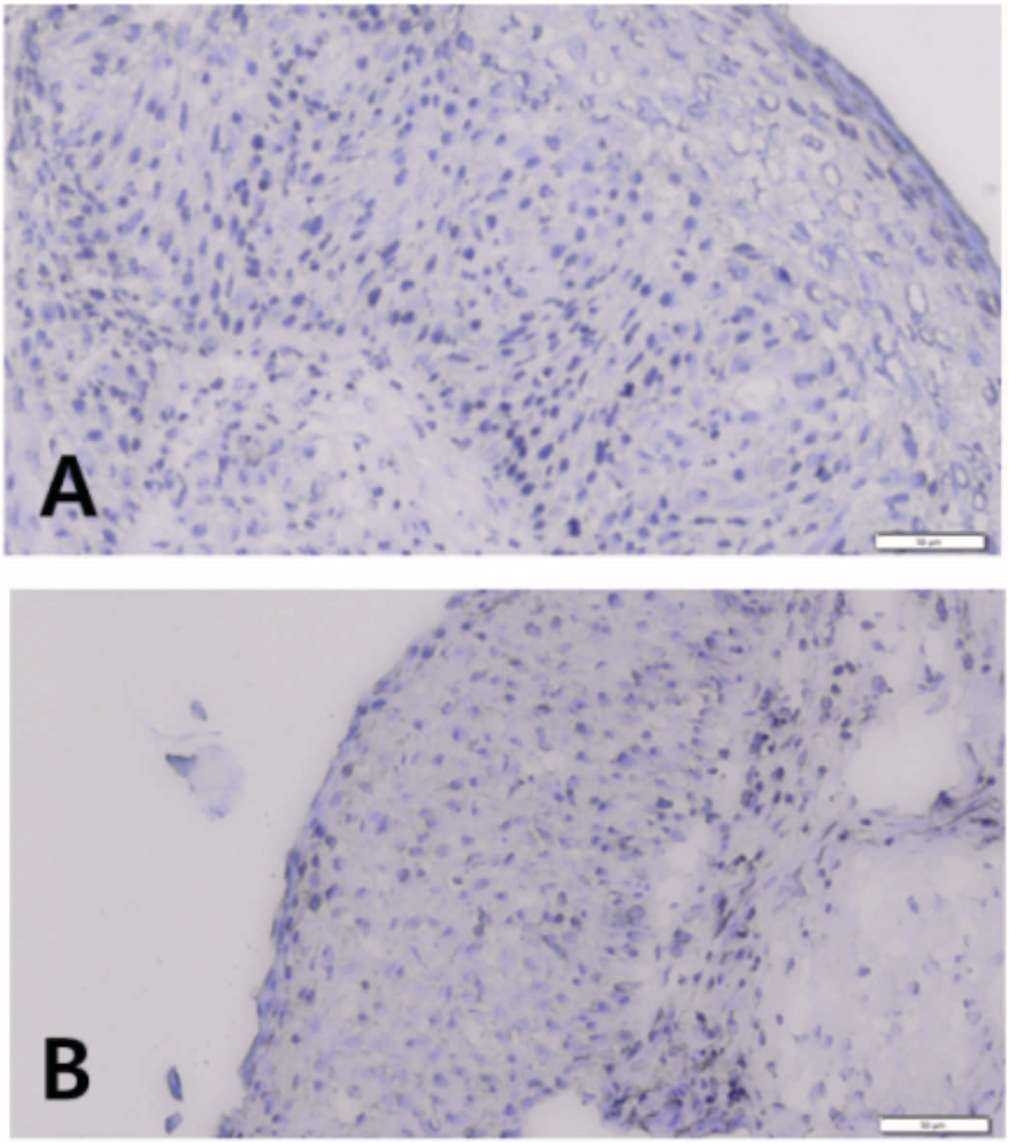

Supporting Information Figure S4: Representative histology of CIN before the microdissection. Unfixed frozen tissues from CIN were cut and lightly stained with hematoxylin. The morphology of neoplastic cells of CIN 2 **(A)** and CIN 3 **(B)** are good enough to be differentiated from nonneoplastic cells.

Supporting Information Table S1: The description of whole-exome sequencing data

Samples*	Sequencing reads	Mapped (%)	Coverage (mean)**	% of bases (>= 20 reads)**
CIN_5N	66,310,825	64,482,875 (97.2%)	84.51	87.1
CIN_5T	49,135,258	48,044,097 (97.8%)	61.34	83.2
CIN_6N	47,709,875	46,574,916 (97.6%)	60.45	83.0
CIN_6T	63,541,303	62,012,386 (97.6%)	80.24	86.2
CIN_9N	54,638,730	53,317,826 (97.6%)	67.95	83.4
CIN_9T	56,763,081	55,360,723 (97.5%)	70.89	83.8
MIC_1N	52,736,101	51,777,623 (98.2%)	67.68	82.9
MIC_1T	62,496,250	61,299,398 (98.1%)	79.21	85.8
CSCC_6N	50,658,090	49,944,276 (98.6%)	62.97	81.9
CSCC_6T	54,792,170	54,132,305 (98.8%)	69.30	83.1
CSCC_12N	64,421,217	63,557,387 (98.7%)	80.87	85.9
CSCC_12T	55,893,905	55,231,335 (98.8%)	69.53	82.9
CSCC_13N	60,110,919	59,381,257 (98.8%)	75.25	85.0
CSCC_13T	54,925,765	54,234,625 (98.7%)	68.56	81.9
CSCC_18N	52,090,557	51,422,761 (98.7%)	64.52	81.8
CSCC_18T	59,114,388	58,374,984 (98.8%)	74.07	83.4

*The neoplasia and matched normal genomes are discriminated with the use of 'T' and 'N', respectively.
**The mean coverage and the % of bases (>= 20 reads) were calculated onto the targeted regions (Agilent SureSelect 50Mb exon).

Supporting Information Table S2: Somatic point mutations and indels identified across eight cervical neoplasia genomes

Supporting Information Table S3: Copy number alterations identified across eight cervical neoplasia genomes

Supporting Information Table S4: Recurrent CNA regions

Position	CytoBand	Event	CIN (N=3)	MIC/CSCC (N=5)	CancerGene*
1:144,009,907–237,465,552	q21.1–q43	Gain	0	4	*PDE4DIP, BCL9, ARNT, TPM3, MUC1, PRCC, NTRK1, SDHC, FCGR2B, PBX1, ABL2, TPR, MDM4, ELK4, SLC45A3, H3F3A, FH*
2:232,656,217–234,233,938	q37.1	Loss	1	2	
3:0–88,914,346	p26.3–p11.1	Loss	0	4	*SRGAP3, FANCD2, VHL, PPARG, RAF1, XPC, MLH1, MYD88, CTNNB1, SETD2, BAP1, PBRM1, FHIT, MITF, FOXP1*
3:112,344,459–198,022,430	q13.2–q29	Gain	0	4	*GATA2, RPN1, FOXL2, WWTR1, GMPS, MLF1, EVI1, PIK3CA, SOX2, ETV5, EIF4A2, BCL6, LPP, TFRC*
4:4,244,789–49,063,301	p16.3–p11	Loss	1	2	*SLC34A2, PHOX2B*
4:87,413,472–87,591,066	q21.3	Loss	0	3	
4:98,447,690–98,964,444	q22.3–q23	Loss	0	3	
6:170,299,004–171,115,067	q27	Loss	1	2	
11:73,147,423–135,006,516	q13.4–q25	Loss	0	5	*PICALM, MAML2, BIRC3, ATM, DDX10, POU2AF1, SDHD, PAFAH1B2, PCSK7, MLL, DDX6, CBL, ARHGEF12, FLI1*
19:320,894–2,293,032	p13.3	Loss	1	2	*FSTL3, STK11, TCF3, GNA11, SH3GL1*

*Cancer Gene census: http://www.cancer.sanger.ac.uk/cosmic/census

Supplementary Table S5: Gene ontology analysis of mutations using DAVID

Supplementary Table S6: Mutations that co-occurred with CNAs
